# Nutrition profile and potency of RGD motif in protein hydrolysate of green peas as an antifibrosis in chronic kidney disease

**DOI:** 10.22038/ijbms.2021.50291.11459

**Published:** 2021-06

**Authors:** Meilinah Hidayat, Sijani Prahastuti, Muhammad Yusuf, Khomaini Hasan

**Affiliations:** 1 Faculty of Medicine, Universitas Kristen Maranatha, Jalan Prof. Drg. Suria Sumantri 65 Bandung 40163, Indonesia; 2 Research Center for Molecular Biotechnology and Bioinformatics, Universitas Padjadjaran, Jalan Singaperbangsa No. 2, Bandung 40133, Indonesia; 3 Faculty of Medicine, Universitas Jenderal Achmad Yani, Jalan Terusan Jenderal Sudirman, Cibeber, Kec. Cimahi Selatan, Cimahi 40531, Indonesia

**Keywords:** Convicilin protein, Fibronectin, Fibrosis, Peas, Pisum sativum, Protein hydrolysates, RGD motif, TGF-Beta 1

## Abstract

**Objective(s):**

Fibrosis is the major cause of chronic kidney injury and the primary etiology in diabetic glomerulosclerosis. The initial study of protein hydrolysate of green peas hydrolyzed by bromelain (PHGPB) considered it to improve kidney function parameters and showed no fibrosis in histopathology features in gentamicin-induced nephrotoxicity rats. In the current study, we aimed to assess the nutrition profile and potency of RGD in PHGPB as antifibrosis in chronic kidney disease (CKD).

**Materials and Methods:**

Green peas (*Pisum sativum*) were hydrolyzed by bromelain from pineapple juice to obtain PHGPB. The amino acid content of PHGPB was measured using the UPLC method, while the primary structure used LC-MS/MS. Bioinformatic analysis was conducted using the Protease Specificity Predictive Server (PROSPER). The potency of RGD in PHGPB was characterized by determining the levels of Fibronectin (FN) and TGF-β1 in mesangial SV40 MES 13 cell lines of diabetic glomerulosclerosis.

**Results:**

The level of lysine was 364.85 mg/l. The LC-MS/MS data showed two proteins with 4–15 kDa molecular weight originated from convicilin (P13915 and P13919) which were predicted by PROSPER proteolytic cleavage, resulted in RGD in the LERGDT sequence peptide. PHGPB increased SV40 MES 13 mesangial cell proliferation that died from high-glucose levels (diabetic glomerulosclerosis model). PHGPB and RGD reduced the levels of FN and TGF-β1 in mesangial cell lines of diabetic glomerulosclerosis.

**Conclusion:**

The nutrition profile and RGD motif in PHGPB show great potential as antifibrosis in CKD.

## Introduction

In Indonesia, the incidence of chronic kidney disease (CKD) continually increases every year. This disease is characterized by decreasing kidney filtration capability for a long period. Decreased kidney filtration capability is mainly caused by fibrosis in the kidney interstitium. Fibrosis is the principal trait of chronic kidney injury characterized by accumulation of extracellular matrix components that replace damaged tissue (1, 2). Glomerular podocytes are mainly involved in the structural and functional integrity of glomerular filtration (3). Podocytes are terminally differentiated renal glomerular cells and their function is essential for integrity of kidney filters. The genetic and histological data greatly affect podocytes (4). Podocyte injury will influence the initiation and development of diabetic nephropathy (DN). Actin cytoskeleton remodeling and dynamic attachment to the glomerular base membrane through integrins (α3β1, αvβ3) are important in protecting the function of glomerular filters. (3). Therefore, therapies aimed at improving podocyte repairment involving integrins in preventing fibrosis formation seem to have a significant effect on new treatment protocols for patients with DN or CKD.

Integrin is a heterodimeric transmembrane glycoprotein that connects cells and matrix–cell interactions. After binding to ligands in the extracellular matrix (ECM), integrins activate intracellular signaling and affect various cell functions, including proliferation, cell adhesion, and migration as well as ECM homeostasis. Integrin is classified based on its function into three groups, namely, the receptor that binds to laminin, collagen, and arginine–glycine–aspartic acid (RGD) (5). RGD is the amino acid sequence within the extracellular matrix protein Fibronectin (FN) that may use Integrin as receptor for cell adhesion molecules. Some αv integrins are expressed in the kidney and play pivotal roles in the progression and development of renal fibrosis (6). 

Several lines of evidence suggest that integrins are involved in the process of renal fibrosis. Deletion of αv-integrin in Pdgfrb cell subtypes leads to protection against unilateral ureteral obstruction-induced renal fibrosis (6). TGF-β1 also plays a very important role in the occurrence of renal fibrosis, making it the main therapeutic target. Suppressing the activity of TGF-β will prevent chronic fibrotic diseases such as CKD (7) and inhibit inflammation resulting in a proper repair effect (8). Overexpression of TGF-β1 induces renal fibrosis while inhibition of TGF-β1 prevents renal fibrosis in a wide range of disease models (9). These findings indicate that work unity of RGD- FN- integrins, and TGF-β1 may play an essential role in the development of renal fibrosis.

Kidneys are the primary organs that dispose of metabolic waste, especially in the form of nitrogen-based products, including urea and ammonia, which are the end products of protein and amino acid metabolism (10-12). In CKD, the kidney fails to effectively cleanse waste products and other compounds from the blood (13). However, intake of protein needs to be fulfilled for sustainable metabolism in the human body. The solution for protein intake of CKD patients is a provision of protein supplements that do not aggravate the kidney function of the patients. A good source of essential amino acids needs to be obtained, which can meet the body’s need for protein but does not cause podocyte damage. Adequate nutrition plays a crucial role in slowing down the progression of kidney disease and improving the function of the kidney as well as the quality of life of patients with CKD. Protein hydrolysates as an alternative treatment for CKD may come from bioactive peptides in a variety of food proteins such as soybeans, egg whites, milk, rice, royal jelly, fish, and green peas (14).

Nutritionally, peas (*Pisum sativum*) are not classified as a complete protein because their protein content loses at least one essential amino acid. However, peas contain high lysine, a carnitine precursor that is needed to convert fatty acids into energy. Lysine is an essential amino acid that can maintain a healthy immune system. Pea protein powders can potentially be an excellent option for supplemental protein in patients with kidney disease (15).

Several noteworthy studies have shown that vegetarian protein intake obtains a better protective effect against deterioration stages of CKD than animal intake protein (16-18). By reducing the intake of animal protein and replacing it with vegetable protein, it is hoped further renal fibrosis or glomerulosclerosis can be prevented. Our previous study showed that orally feed of 200 mg/kg BW/day of PHGPB for 28 days improves kidney function levels (19) and showed no fibrosis histopathology feature in Cisplatin-induced nephrotoxicity rats (20). The active substance of PHGPB was predicted to support integrins in repairing podocyte injury and fibrosis in a concentration- or dosage-dependent manner.

To clarify the study, the nutrition profile and amino acid content of PHGPB will be determined, sequence active peptides in PHGPB were analyzed using a bioinformatics approach, and potency of RGD in PHGPB on fibronectin (FN) and TGF-β1 levels in glucose-induced mesangial cells as diabetic glomerulosclerosis model will be assessed. This study aimed to assess the nutrition profile and potency of RGD in PHGPB as antifibrosis in CKD. 

## Materials and Methods

Green peas (*Pisum sativum* L.) were purchased commercially from Magelang Plantation, Maica leaf, in Central Java, Indonesia. Bromelain was isolated from pineapple juice (*Ananas sativus*) that was purchased from North Bandung, Subang, Indonesia. RGD (87% purity) was kindly provided by Dr. Rani Maharani (Department of Chemistry, Universitas Padjadjaran). The RGD peptide was synthesized according to Merrifield’s method with some modification (21-23).

***Protease for hydrolysis process***

The obtained pineapple juice was filtered and centrifuged at 2500 g*, *4 °C, for 10 min (Tomy Portable Refrigerated Centrifuge MX-201). Bradford method was used to determine the protein concentration of bromelain (24). Kunitz’s method was used to measure the total specific activity of the enzyme and the content of the hydrolysate protein with bovine serum albumin as a standard (25) and the standard used the tryptophan curve (26). 

***Protein hydrolysate preparation***

A simple hydrolysis method with modification is used to prepare the protein hydrolysates (27). As much as 500 g dry seeds of green peas were mashed, then sieved through a 120MESH sieve and dissolved in water (2000 ml). Then 10% (w/v) bromelain solution was added and left at room temperature (25 °C–30 °C) on a stirrer for 72 hr. The solution was then transferred into a tube and for 10 min was centrifuged at 2500*g, *4 °C. Using a filter paper the supernatant was filtered. The molecular weight of the obtained protein hydrolysates was separated and determined using SDS-PAGE (28).

***The nutrition profile content determination***

The sample was precisely weighed from 0.1 g to 1.0 g into a 20 ml headspace vial and 5 ml of 6 N HCl added. The vial was closed and heated in an oven at 110 °C for 22 hr. The vial was then removed and cooled. The sample solution was filtered twice with paper and GHP/RC syringe filter, respectively. 40 ml of internal amino acid standard was added to the samples and diluted by aquabidest up to a total volume of 500 ml (29, 30). The sample and internal standards were then derivatized and injected to UPLC (ESI-QTOF) using AccQ.tag Ultra C18 1.7 µm column. Analysis of amino acids in PHGPB was carried out with UPLC; while for protein analysis, Kjeldahl (U relative 46%, EKP 0.13); for fat, the Weibull method; and for carbohydrates the glucose oxidase methods were used. The results are shown in Table 1. The formula for calculating PDCAAS percentage is as follows: the amount of limiting amino acid (in mg) in 1 g of test protein/mg of the same amino acid of the reference protein in 1 g) × percentage of fecal true digestibility. Soybean protein is used as a reference protein. The fecal true digestibility percentage of peas (*P. sativum*) is 97% (31). 

***Protein hydrolysate of green peas primary structure determination***

The sample was obtained from in-gel digestion (Manual Instructions, In Gel Tryptic Digestion, Pub. No. MAN0011497, Thermo Scientific). The primary structure of PHGPB was partially determined by LC-MS/MS (Thermo Q-Exactive Mass Spectrometry) (User Guide: Pierce C18 Spin Columns, 2011, Thermo Scientific) by using the database of *P. sativum *(Taxonomy ID: 3888). Data were analyzed using Proteome Discoverer 2.1 software (Align Tools, Uniprot. Cited February 6, 2019. Available from https://www.uniprot.org.align). 

***Prediction of active peptide sequences using bioinformatics approach***

Protease specificity prediction servers (PROSPER) were used to predict the proteolytic sites of bromelain against PHGPB (http://lightning.med.monash.edu.au/PROSPER/). In this process, the specificity of the peptide model substrate will be obtained, which is assumed as an active substance in the PHGPB mechanism as an antifibrosis (32). 

***Glucose-induced mesangial cells for proliferation and viability assay***

This viability test is performed to assess the toxic effect of PHGPB and determine its effect on mesangial kidney cell proliferation. The results of this test are used to determine the dose that will be used in the effectiveness test on the FN and TGF-β1 parameters.

The SV40 MES 13 ATCC® CRL-1927™ cell line of *Mus musculus *glomerular mesangial kidney was obtained from Aretha Medika Utama, Biomolecular and Biomedical Research Center, Bandung, Indonesia. Using the MTS (3-(4,5-dimethylthiazol-2-yl)-5-(3-carboxymethoxyphenyl)-2-(4-sulfophenyl)-2Htetrazolium) Proliferation Assay Kit (Abcam®, ab197010) the viability assay was performed (33). In brief, 5×10^3^ cells per well were seeded in F12-K medium (Gibco, 10270106) fetal bovine serum (FBS), supplemented with 1% antimycotic- antibiotic (Gibco, 1772653) and 1% HEPES (Sigma Aldrich, 1002184736). In a 96-well plate (Corning, 3596) the cells were cultured and at 37 °C were incubated for 24 hr under 5% CO_2_. The medium was replaced with a fresh medium (180 μl), to which was added 20 μl of PHGPB (25 and 100 µg/ml) and 10% DMSO in triplicate. The plates were incubated for 24 hr at 37 °C under 5% CO_2_; Untreated cells were used as control. To each well was added 20 μl of MTS. The plate was incubated for 4 hr at 37 °C under 5% CO_2_. Absorbance was recorded at 490 nm by using a Multiscan GO (Thermo Scientific, U.S.A) plate reader (33, 34). In brief, 5×10^3^ SV40 MES 13 cells ATCC® were plated in a 96-well plate with growth medium (200 μl) and incubated for 24 hr at 37 °C under 5% CO_2_; then the medium was discarded. To the cells were added 180 μl of 0, 10, and 20 mM glucose-induced medium, 20 μl of PHGPB (25 and 100 µg/ml), and RGD (87% purity, 25 and 100 µg/ml). The cells were incubated for 14 days at 37 °C under 5% CO_2_. (Abcam, ab197010). Absorbance at 490 nm was recorded using the Multiscan GO (Thermo Scientific, U.S.A) plate reader to calculate mortality percentage (34, 35). 

***Glucose induction in mesangial cells for measurement of fn and TGF-β1 levels***

Confluent 80% cell cultures were washed two times with 1 ml of 1X PBS. Then trypsin-EDTA (1 ml) was added to the cells and incubated for 3 min until the cells were released. The cells were transferred into a Falcon tube containing the culture medium (5 ml) and centrifuged for 5 min at 500 g. Then, the supernatant was removed and the cells were resuspended with the culture medium (1 ml). Using a hemocytometer the cells were counted. Moreover, 100,000 cells per well were plated on 6-well plates with the medium (2 ml) then incubated for 24 hr in an incubator under 5% CO_2 _at 37 °C. The medium was removed and replaced with 2 ml of 20 mM and 75 mM glucose induction medium. To the sample was added 25 or 100 µg/ml PHGPB and incubated for 14 days at 37 °C and 5% CO_2_. The doses used were chosen based on the cytotoxic test results. The maximum concentration range applied to cytotoxicity assays for reference compounds was 100 μM (36). The cell medium was then collected for testing, and the levels of FN and TGF-β1 were determined using the ELISA method (34, 35). 

***Measurement of FN and TG-β1 levels***

To each well was added 100 µl of the capture antibody and incubated at 4 °C overnight. The plate was washed using 200 µl of wash buffer four times, and to each well was added the assay diluent (200 µl). Then the plate was sealed and incubated at 200 rpm and room temperature for 1 hr. The plate was washed using 200 µl of wash buffer four times. To the wells for the standard was added the standard (100 µl), and to those for the samples was added the sample (100 µl). The plates were then incubated at 200 rpm and room temperature for 2 hr. The plate was washed using 200 µl of wash buffer four times. To each well was added 100 µl of detection antibodies. Then the plate was sealed and incubated at 200 rpm and room temperature for 1 hr. The plate was washed using 200 µl of wash buffer four times. To each wall was added with 100 µl of HRP–avidin. The plate was then sealed and incubated for 30 min at 200 rpm and room temperature. The plate was washed using 200 µl of wash buffer five times. To each well was added TMB substrate (100 µl) and incubated in the dark for 15–30 min. In the last step, the stop solution (100 µl) was added. Absorbance was read at 450 nm (37, 38). 

***Statistical analysis of FN and TGF-β1 levels***

To verify the results of diﬀerent treatments data were analyzed using one-way analysis of variance (ANOVA) on SPSS software (SPSS, Chicago, Illinois) version 16. To validate significant diﬀerences *post hoc* Tukey HSD and *t*-test were used (α< 0.05).

## Results

***Nutrition profile and amino acids content in PHGPB***

Based on UPLC measurement, PHGPB contains 19.08 kcal/ 100 g, low carbohydrates, low fat, 3.03% protein, and several high levels of amino acids, especially Lysine (36.58%). Complete results of macronutrient profile, and 18 amino acids content in PHGPB are presented in Table 1. 

Protein Digestibility Converted Amino Acid Score (PDCAAS) of PHGPB was calculated as 36.58/35× 0.97= 1.045.

***Characteristics of PHGPB by LC-MS/MS***

Based on SDS PAGE analysis PHGPB contains molecular weights of < ~4 and 4–15 kDa and based on analysis result using LC-MS/MS, only one protein was identified from the protein band with a molecular weight of less than 4 kDa. The protein is indicated as elongation factor 1-alpha. From the protein band with a molecular weight of 4–15 kDa, two proteins are identified which belong to convicilin. The LC-MS/MS results of PHGPB are shown in Table 2.

**Figure 1:**
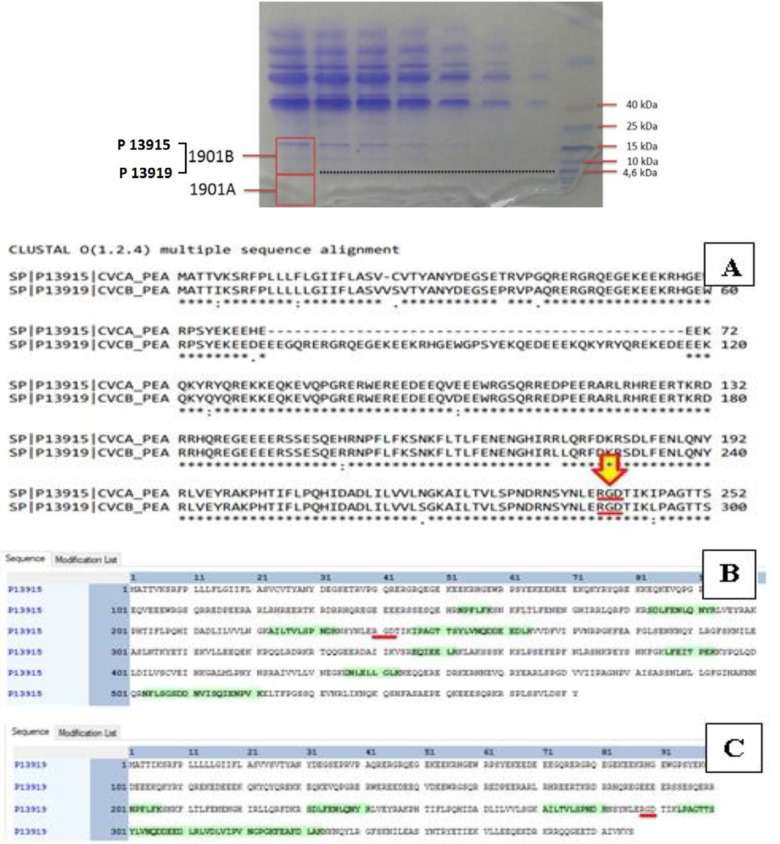
SDS-PAGE Electroforegram from PHGPB. Two boxes at the left show excised region for samples 1901A and 1901B. In region 1901B there are two proteins, P13915 and P13919. The Location of RGD peptide in the whole sequence of protein 1901B. A. The Location of RGD in elongation factor 1-alpha Protein Convicilin of P13915 and P13919 (red arrow, red underline) in lines 252 and 300, respectively. B. The location of RGD peptide identified from protein convicilin P13915 (red underline) in line 201, column 41. C. The location of RGD peptide identified from protein convicilin P13919 (red underline) in line 201, between columns 81 and 91

Two proteins were identified from the SDS PAGE sample (molecular weight 4-15 kDa) with a similar name (Convicilin) but with a different accession number, i.e., P13915 and P13919. Convicilin P13915 consisted of 571 amino acid residues, meanwhile, Convicilin P13919 consisted of 386. Both convicillins have high similarities in amino acid sequence as shown in alignments result between convicilin P13915 and P13919. The locations of the RGD peptide in the whole sequence of protein 1901B, convicilin P13915, and convicilin P13919 are shown in Figure 1.

***Prosper analysis of PHGPB convicilin P13919***

**Figure 2:**
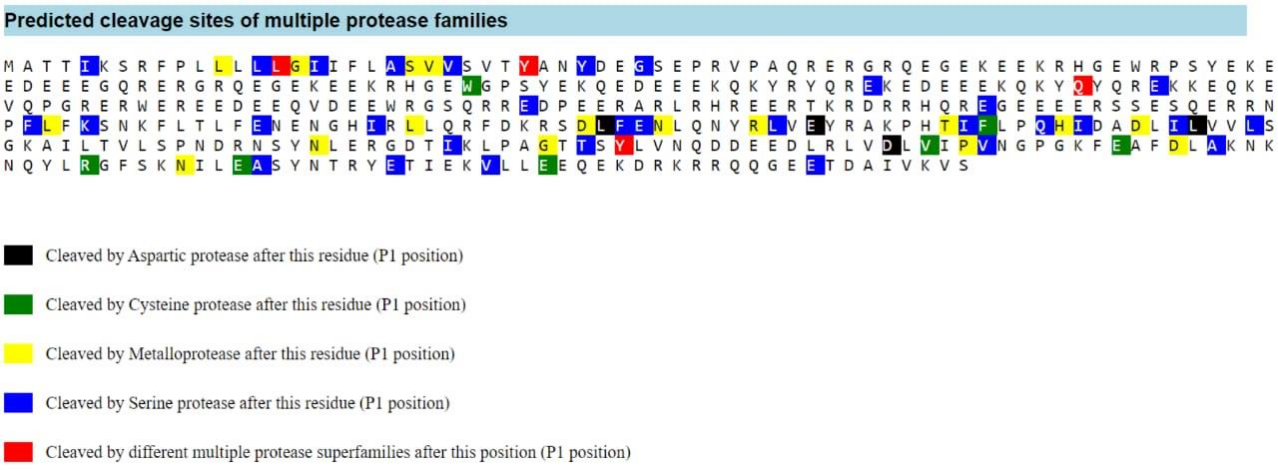
The predicted cleavage sites of multiple protease families in protein convicilin P13919 by PROSPER. Black: (P1 position) after this residue cleaved by aspartic protease, Green: (P1 position) after this residue cleaved by Cysteine protease, Yellow: (P1 position) after this residue cleaved by Metalloprotease, Blue: (P1 position) after this residue cleaved by Serine protease, Red: (P1 position) after this position cleaved by different multiple proteases. Location of RGD cleavage sites showed as LERGDT between cleavage by metalloprotease and serine protease (underlined)

**Figure 3:**
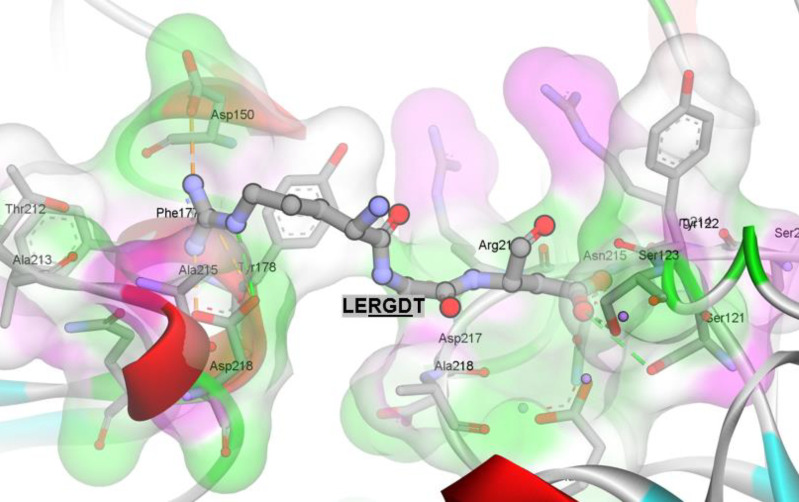
Prediction of arginine-glycine-aspartate (Arg- Gly- Asp) (RGD) in leucine-glutamate-arginine-glycine-aspartate-threonine (LERGDT) binding as integrin ligand

Analysis of proteolytic cleavage by metalloprotease and serine protease predicted that RGD results in a short peptide LERGDT (Figure 2). The predicted cleavage sites of RGD in convicilin P13919 and its interaction with the extracellular segment of integrin αvβ3 are shown in Figure 3 (Patent P00201907647 Republic of Indonesia) (39). 

***Cytotoxic test results on mesangial cells for PHGPB***

The viability of glucose (10 and 20 mM)-induced SV40 MES 13 ATCC® cells treated with PHGPB (25 and 100 μg/ml) during 15 days of incubation were demonstrated in Table 3. Both doses of PHGPB treatments showed a high level of viability (> 90%) and significant differences compared with control (*P*<0.05). The results of the cytotoxic test with or without glucose induction showed that both concentrations were safe; thus, 25 and 100 μg/ml in 0,10, and 20 mM glucose were used.

On day 15, high viability was found in all PHGPB-treated cells. In 0 mM glucose induction, it appears that the highest viability was in PHGPB (25 µg/ml) (139.75±5.3 %) followed by PHGPB (100 µg/ml) with 10 mM glucose induction (137.13±6.70%). In 20 mM glucose induction, observations showed the highest viability was seen in a PHGPB dose of 100 µg/ml (96.47±5.19%). The PHGPB dose of 100 µg/ml increased the viability of 20 mM of glucose-induced mesangial cells by 1.48 times compared with control. 

***The results of the fibronectin in mesangial cells after PHGPB and RGD treatment***

**Figure 4:**
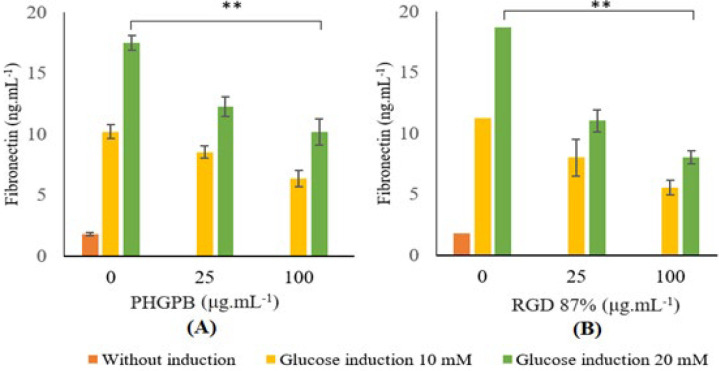
Treatment effects of (A) protein hydrolysate of green peas bromelain (PHGPB) and (B) arginine-glycine-aspartate (Arg- Gly- Asp) (RGD) 87% on fibronectin level in SV40 cell MES 13 with glucose (10 and 20 mM) induction

Glucose induction in SV40 MES 13 cells showed increased levels of FN. Administration of PHGPB or 87% RGD on SV40 MES 13 cells reduced levels of FN (Figure 4).

The mesangial cells without PHGPB treatment, showed high level of FN (Figure 4A) (glucose 10 nM: 10.20 ± 0.60^c^ ng/ml; glucose 20 nM: 17.48 ± 0.62^e^ ng/ml). Provision of both doses of PHGPB showed decreased FN levels. In low dose glucose, (glucose 10 nM: 25 μg/ml PHGPB: 8.51 ± 0.52^c ^ng/ml; 100 μg/ml PHGPB: 6.35 ± 0.67^b ^ng/ml, 37.74% lower than positive controls, (*P*<0.05)), PHGPB 25 showed no significant difference with control while PHGPB 100 showed highly significant difference from control. In high dose glucose, (glucose 20 nM: 25 μg/ml PHGPB: 12.25 ± 0.80^d ^ ng/ml; 100 μg/ml PHGPB: 10.15 ± 1.08^c^ ng/ml, 41.93% lower compared with positive controls (*P*<0.05)), PHGPB 25 showed significant difference with control while PHGPB 100 showed highly significant difference from control, marked with two stars symbol (Figure 4). Provision of higher dose of PHGPB tends to obtain lower FN level. 

The mesangial cells without RGD treatment, showed high level of FN (glucose 10 nM: 11.25 ± 1.51^c^ ng/ml; glucose 20 nM: 18.69 ± 0.91^d ^ng/ml) (Figure 4B). Provision of both doses of RGD showed decreased FN levels. In low dose glucose -induce, (glucose 10nM: 25μg/ml RGD: 7.99 ± 0.61^b^ ng/ml; 100 μg/ml RGD: 5.54 ± 0.21^b^ ng/ml, 50.75% lower than positive controls, (*P*<0.05)), both doses of RGD showed highly significant difference from control. In high dose glucose-induce, (glucose 20 nM: 25 μg/ml RGD: 11.01 ± 0.54^c^ ng/ml; 100 μg/ml RGD: 8.02 ± 1.54^b^ ng/ml, 57.08% lower than positive controls (*P*<0.05)), both doses of RGD showed highly significant difference from control. Provision of a higher dose of RGD showed a lower FN level. However, the provision of RGD in high glucose induce showed a better result in lowering the FN level than the provision of PHGPB (57.08% and 41.93%, respectively).

***The results of the TGF-β1 levels in mesangial cells after PHGPB and RGD treatment***

**Figure 5:**
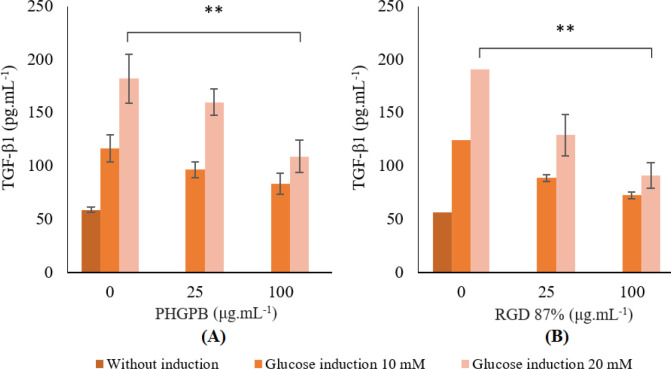
Treatment effects of (A) protein hydrolysate of green peas bromelain (PHGPB) and (B) arginine-glycine-aspartate (Arg- Gly- Asp) (RGD) 87% on TGF-β1 level in SV40 cell MES 13 with glucose (10 and 20 mM) induction

Glucose induction in SV40 MES 13 cells showed increased levels of TGF-β1. Administration of PHGPB or 87% RGD on SV40 MES 13 cells reduced levels of TGF-β1 (Figure 5).

The mesangial cells without PHGPB treatment, showed high level of TGF-β1 (Figure 5A) (glucose 10 nM: 116.33± 12.54^b^ pg/ml; glucose 20 nM: 181.97± 22.96^c^ pg/ml). Provision of both doses of PHGPB showed decreased TGF-β1 level. In low dose glucose-induce (glucose 10 nM: 25 μg/ml PHGPB: 96.50± 7.48^b^ pg/ml; 100 μg/ml PHGPB: 83.14± 9.68^ab^ pg/ml) neither dose, PHGPB 25 and 100, showed any significant difference with control. In SV40 MES 13 cells high-dose-glucose induce (glucose 20 nM: 25 μg/ml PHGPB: 159.89± 12.40^c^ pg/ml; 100 μg/ml PHGPB: 108.75± 15.18^b^ pg/ml, 40.23% lower (*P*<0.05) compared with positive controls), PHGPB 25 showed significant difference with control while PHGPB 100 showed highly significant difference from control, marked with two stars symbol (Figure 5). Provision of higher dose of PHGPB showed lower TGF-β1 level. 

The mesangial cells without RGD treatment, showed high level of TGF-β1 (Figure 5B) (glucose 10 nM: 124.35± 3.34^c^ pg/ml; glucose 20 nM: 190.51± 19.34^d^ pg/ml) (Figure 4). Provision of both doses of RGD showed decreased TGF-β1 level. In low glucose (glucose 10 nM: 25 μg/ml RGD: 88.52± 3.09^ab^ pg/ml; 100 μg/ml RGD: 90.75 ± 14.17^b^ pg/ml), RGD 25 showed significant difference from control while RGD 100 showed highly significant difference from control. In high dose glucose, (glucose 20 nM: 25 μg/ml RGD: 128.84 ± 12.05^c^ ng/ml; 100 μg/ml RGD: 90.75 ± 14.17^b^ pg/ml, 52.36% (*P*<0.05) lower than positive controls (*P*<0.05)), both doses of RGD showed highly significant difference from control. Provision of higher dose of RGD showed lower TGF-β1 level.

The provision of RGD in high-glucose-induced showed a better result in lowering the FN level than PHGPB (52.36% and 40.23%, respectively).

**Table 1:** Analysis of macronutrients and amino acids in protein hydrolysate of green peas bromelain (PHGPB)

**No.**	**Parameter**	**Result simplo**	**Result duplo**	**Method**
1	Total Energy (kcal/100mL)	17.96	19.08	Calculation
2	Carbohydrate (%)	1.55	1.74	Glucose Oxidase
3	Protein (%)	2.94	3.03	Kjeltec/Kjehldahl
4	Total Fat (%)	< 0.02	< 0.02	Weilbull
5	Water Content (%)	94.95	94.67	SNI 01-2891 - 1992, point5 . 1
6	Ash Content (%)	0.56	0.56	SNI 01-2891-1992, 6.1
7	L-Tryptophan (mg / L)	202.45	198.67	HPLC
8	L-Methionine (mg / L)	-	-	UPLC
9	L-Lysine (mg / L)	366.35	365.30	UPLC
10	L-Histidine (mg / L)	258.33	258.97	UPLC
11	L-Serine (mg / L)	327.44	329.00	UPLC
12	L-Arginine (mg / L)	295.69	297.18	UPLC
13	Glycine (mg / L)	431.98	433.68	UPLC
14	L- Aspartic acid (mg / L)	715.47	718.00	UPLC
15	L- Glutamic acid (mg / L)	1454.85	1457.28	UPLC
16	L-Threonine (mg / L)	280.04	281.08	UPLC
17	L-Alanine (mg / L)	325.42	326.33	UPLC
18	L-Proline (mg / L)	322.74	322.52	UPLC
19	L-Cystine (mg / L)	24.52	24.64	UPLC
20	L-Phenylalanine (mg / L)	395.32	395.46	UPLC
21	L-Tyrosine (mg / L)	132.16	132.59	UPLC
22	L-Valine (mg / L)	393.62	393.03	UPLC
23	L-Isoleusine (mg / L)	319.88	320.86	UPLC
26	L-Leusine (mg / L)	484.42	485.08	UPLC

UPLC: Ultra Performance Liquid Chromatography

**Table 2:** Results of LC-MS/MS analysis of protein hydrolysate of green peas bromelain (PHGPB) with molecular weight less than ~4 kDa – 15 kDa

**Protein MW less than ~4 kDa** **1901A**		**Protein MW 4-15 kDa** **1901B**	
Accession Description(*)	Q41011 Elongation factor 1-alpha [OS=*Pisum sativum*]	**P13915** Convicilin [OS=*Pisum sativum*]	**P13919** Convicilin [OS=*Pisum sativum*]
Number peptides identified	3	8	6
Sequence for eachpeptide identified	1. QTVAVGVIK 2. IGGIGTVPVGR 3. LPLQDVYK	1. GNLELLGLK 2. SDLFENLQNYR 3. NFLSGSDDNVISQIE NPVK 4. AILTVLSPNDR 5. NPFLFK 6. IPAGTTSYLVNQDD EEDLR 7. EQIEELR 8. LFEITPEK	1. SDLFENLQNYR 2. LVDLVIPVNGPGK 3. AILTVLSPNDR 4. NPFLFK 5. IPAGTTSYLVNQDDE EDLR 6. FEAFDLAK

*Data analysis using Proteome Discoverer 2.1 software

**Table 3:** Cytotoxic effect of protein hydrolysate of green peas bromelain (PHGPB) towards mesangial cells without and with Glucose induction

**Sample**	**Cell viability (%)**
Control glucose induction 0 mM	100.00±4.93^cd^
Control glucose induction 10 mM	77.14±0.65^ab^
Control glucose induction 20 mM	65.09±6.41^a^
PHGPB 25 ug/ml, glucose induction 0 mM	139.75±5.37^g^
PHGPB 100 ug/ml, glucose induction 0 mM	121.34±10.70^ef^
PHGPB 25 ug/ml, glucose induction 10 mM	115.31±3.39^de^
PHGPB 100 ug/ml, glucose induction 10 mM	137.13±6.70^fg^
PHGPB 25 ug/ml, glucose induction 20 mM	89.73±3.13^bc^
PHGPB 100 ug/ml, glucose induction 20 mM	96.47±5.19^c^

* Data were presented as means±standard deviation

Lower case differences indicate data significance at* P*<0.05 (Tukey HSD *post hoc* test)

RGD formed hydrogen bonds with Asp150, Asp218, Ser121, and Ser123. Prediction of LERGDT binding as integrin ligand, interaction of RGD with the extracellular segment of integrin αvβ3

## Discussion

PHGPB contains a high level of amino acid, especially Lysine (36.58%) while the reference protein, soybean contains 35% of Lysine (40). Lysine is a limiting essential amino acid (31) that is suitable to meet the protein needs of CKD patients without producing waste that is toxic to the body. A protein-restricted diet supplemented with keto analogs effectively improves kidney endpoints, blood pressure levels, including preserving eGFR, diminishing proteinuria, and CKD-mineral bone disorder parameters without causing side effects malnutrition (41). 

Patients with CKD must meet their required essential amino acid intake and minimize the amino-nitrogen protein. Supplementation of keto analogs of essential amino acids will improve the quality of protein in protein-restricted renal diets without burdening the kidney (42). This keto-analog is transaminated by the enzyme aminotransferases into amino acids corresponding to the way nitrogen is inserted from amino groups derived from the degradation of endogenous amino acids. Consequently, the resulting nitrogen will be less, and the workload of the kidneys will be reduced. (43). 

Few amino acid keto analog supplements, such as ketosteril, are given to patients with CKD. The ketosteril contains several essential amino acids, such as L-threonine, L-tryptophan, L-histidine, L-tyrosine, and L-lysine (44). Ketosteril can provide the needs of amino acids and reduce the intake of amino nitrogen. This drug utilizes nitrogen from amino acids that are not needed to be converted to amino acids to reduce urea synthesis and prevent the accumulation of uremic toxins. Once absorbed, keto acid and hydroxy will bind to the corresponding essential amino acids and then take nitrogen from non-essential amino acids, thus reducing the formation of urea^ (^^45^^).^ PHGPB can supply amino acid needs in impaired kidney conditions since the amino contained in hydrolysate proteins is easily absorbed because the peptide has a small molecular weight.

The PDCAAS value of PHGPB is obtained from the calculation of the amino acid Lysine levels compared with standard amino acid lysine levels, in this case, soybean. The value obtained from the calculation results is greater than 1.00; The PDCAAS of PHGPB values ​​of more than 1.00 are classified as good and easy-to-digest amino acids (31). 

Protein is closely related to kidney diseases and excessive protein intake can harm the kidneys of CKD patients. High protein food intake causes an increase in glomerular filtration rate (GFR), causing ‘glomerular hyperfiltration’, which in turn leads to ‘afferent’ arteriolar dilatation and increased intraglomerular pressure. If this continues for a long time, the podocyte injury will trigger chronic inflammation which results in fibrosis formation (46).

It is interesting to note that the RGD peptides are present in PHGPB convicilin P13915 and P13919. Therefore, the potency of RGD regarding integrin, FN, and TGF-β1 was examined in this study. The high level of viability (> 90%) indicates that PHGPB in the concentration of 100 µg/ml can protect the renal cells from damage caused by glucose induction (Table 3). Results showed that all treatments of PHGPB and RGD 87% reduce the levels of FN and TGF-β1 in the mesangial cell line of diabetic glomerulosclerosis, and significantly different from Control (*P*<0.05) (Figure 4 and 5). Nevertheless, the provision of RGD in high-glucose-induced showed better results in lowering FN and TGF-β1 levels than provision of PHGPB. These findings prove two points, first that RGD in PHGPB has an inhibiting potency of the FN-the integrin-TGF-β1 pathway for formation of fibrosis in renal mesangial cells of the CKD model. Secondly, RGD 87% purity is more potent in lowering FN and TGF-β1 levels because it contains pure and more RGD motif than PHGPB.

FN is recognized by αvβ1 and αvβ3 integrins. The primary sequence motif for integrin binding of FN is a tripeptide, namely, Arg-Gly-Asp (RGD) (47). According to Patent US20120003201A1, the composition substance for treating CKD consists of recycled RGD, which contains a peptide agent associated with binary dome nanoparticle delivery vehicles. RGD integrin is a recognized therapeutic target for thrombosis, fibrosis, and cancer (48). Some αv integrins are expressed in the kidney and play important roles in the development and progression of kidney fibrosis (5). Many noteworthy findings implicate integrins in the pathophysiology of acute kidney injury (AKI) (49, 50). Chronic effects study of RGD peptides in ischemic ARF in rats showed good results. Goligorsky *et al*. study showed a significant difference in creatinine clearance (Cr) three days after nephrectomy surgery, between Sprague-Dawley rats treated with RGDDFLG or RGDDFV cyclic with a comparison control group (RDADFV cyclic). This suggested that RGD peptides may prevent kidney tubular obstruction in the ischemic model of AKI (51).

The RGD peptide prevents and/or repairs glomerular pathological conditions related to DN and other forms of CKD by inhibiting the interaction among mesangial cells with extracellular matrix proteins. RGD inhibits integrin αvβ1 adhesion with mediated primary mesangial cells, thereby preventing FN formation (49). In the viability test, the provision of PHGPB increased the proliferation of SV40 MES 13 mesangial cells that died from high-glucose levels (diabetic glomerulosclerosis model). Therefore, PHGPB exhibits a beneficial effect on glomerulosclerosis. 

FN and TGF-β1 are used as a parameter correlated with glomerulosclerosis because of their existence in glomerulosclerosis disease. TGF-β1 plays an important role in mesangial matrix expansion. The injured glomeruli resulted in TGF-β1 messenger ribonucleic acid (mRNA) expression and TGF-β1 synthesis and also secreted a great amount of FN. This injury in glomeruli can lead to renal disease (52, 53). Increased FN and TGF-β1 levels in SV40 cells after being induced with glucose indicates glomerular damage has occurred. However, after treatment with PHGPB (25 and 100 µg/ml), FN and TGF-β1 levels decreased. 

Wang *et al*. 2008 study, showed that glucose is a potent inducer to increase the FN level, which is the principal matrix protein that accumulates excessively in kidney fibrosis disease (54). Treatment of 100 mg/ml PHGPB on mesangial cells induced with 20 mM glucose lowered TGF-β1 level 40.23% lower than the positive control (*P*<0.05), while the result of 87% RGD treatment, was 52.36% lower than the positive control (*P*<0.05). Therefore, PHGPB has been assumed to have a beneficial effect in hindering the TGF-β1 level, although the result is lower than the potency of RGD with 87% purity. In the process of activation of TGF-β1, it is necessary to bind αv integrin to a sequence of RGD in the prodomain (55). Small molecule inhibitors, such as the RGD-motif in PHGPB, may behave potentially effectively at dampening TGF-β1 signals, which are master regulators of fibrosis and play important roles in many kidney diseases (56). 

The RGD peptide agent is proven to significantly reduce kidney impairment symptoms, such as albumin excretion and mesangial expansion, in people with type 2 diabetes and mice with Ins2Akita/+type 1 diabetes. The RGD peptide consisting of cysteine-free residues and RGD peptide-dome nanoparticles is approximately three times stronger than unmodified RGD peptides. Cysteine-modified RGD peptides, namely, vault complex-like CGRGDSP, are more efficient than unmodified RGD peptides in preventing the progression of DN in the initial phase and/or decreasing injury in DN. Furthermore, cysteine-modified RGD peptide-vault nanoparticle compositions can significantly enhance the *in vivo* delivery of the modified RGD peptides. The RGD domain activates and harmonizes the α5β1 FN interface and synergies to provide mechanical strength to these bonds (47). 

The PROSPER prediction of RGD peptide-binding shows the integrin ligand interaction of LERGDT (40, 57) with the extracellular segment of integrin α_v_β_3_. Small-molecule inhibitors, such as peptide-RGD in the protein hydrolysate of green peas, might be an effective way of dampening TGF-β1 signaling, which has been implicated in numerous fibrotic kidney diseases (52). 

Provision of a higher dose of both PHGPB or RGD showed lower FN and TGF-β1 levels. Active substances in PHGPB influence integrins in repairing fibrosis, depending on the concentration or dosage used. These reductions in FN and TGF-β1 levels supposedly are associated with the RGD or LERGDT in PHGPB in a dose-dependent manner. 

The LERGDT sequence which is hypothesized as a strong agent to prevent FN formation has not been proven yet because of the limitations of the present study.

As expected, our study revealed the positive effects of PHGPB and RGD87% on FN and TGF-B1 activity in glucose-induced SV 40 MES 13 cells. PHGPB was shown to be as effective as RGD87% in preventing the elevation of FN and TGF-B1 levels, the precursors of fibrosis formation. It can be assumed that the beneficial effects of consuming PHGPB on renal function may be mediated mostly by its RGD peptide through its antifibrotic activity.

## Conclusion

The nutrition profile and RGD motif in PHGPB show great potential as antifibrosis in CKD.
